# High targeted migration of human mesenchymal stem cells grown in hypoxia is associated with enhanced activation of RhoA

**DOI:** 10.1186/scrt153

**Published:** 2013-01-07

**Authors:** Grigory Vertelov, Ludmila Kharazi, M G Muralidhar, Givon Sanati, Timothy Tankovich, Alex Kharazi

**Affiliations:** 1 'Stemedica' Cell Technologies, 5375 Mira Sorrento Place, Suite 100, San Diego, CA 92121, USA

## Abstract

**Introduction:**

A feature which makes stem cells promising candidates for cell therapy is their ability to migrate effectively into damaged or diseased tissues. Recent reports demonstrated the increased motility of human mesenchymal stem cells (hMSC) grown under hypoxic conditions compared to normoxic cells. However, the directional migration of hMSC cultured in hypoxia has not been investigated. In this study we examined the *in vitro *transmembrane migration of hMSC permanently cultured in hypoxia in response to various cytokines. We also studied the involvement of RhoA, a molecule believed to play an essential role in the migration of MSC via reorganization of the cytoskeleton.

**Methods:**

We compared the directional migration of human hMSCs grown permanently under normal (21%, normoxic) and low O_2 _(5%, hypoxic) conditions until passage 4 using an *in vitro *transmembrane migration assay. A series of 17 cytokines was used to induce chemotaxis. We also compared the level of GTP-bound RhoA in the cell extracts of calpeptin-activated hypoxic and normoxic hMSC.

**Results:**

We found that hMSC cultured in hypoxia demonstrate markedly higher targeted migration activity compared to normoxic cells, particularly towards wound healing cytokines, including those found in ischemic and myocardial infarction. We also demonstrated for the first time that hMSC are dramatically more sensitive to activation of RhoA.

**Conclusions:**

The results of this study indicate that high directional migration of hMSCs permanently grown in hypoxia is associated with the enhanced activation of RhoA. The enhanced migratory capacity of hypoxic hMSC would further suggest their potential advantages for clinical applications.

## Introduction

Mesenchymal stem cells (MSC) or multipotent stromal cells are non-hematopoietic progenitor cells with potential to differentiate into multiple lineages - adipogenic, osteogenic and chondrogenic. They are known to possess many features which make them an attractive candidate for stem cell therapy or drug delivery applications. Specifically, it has been established that MSC are capable of self-renewal [1], secreting a wide spectrum of cytokines and factors [2], and also have a unique ability to fuse with damaged cells [3-5]. Furthermore, some data indicate that MSC are able to migrate *in vivo *to the site of injury [6-8].

It is important to preserve these characteristics during tissue culture expansion, a necessary step towards the generation of clinically significant cell numbers. One of the major complications at this step is spontaneous cell differentiation, which can occur particularly in the presence of bovine serum. A possible approach to alleviate this problem is cultivation of MSC in a low oxygen environment. The underlying premise is that *in vivo *MSC reside in an environment with a relatively low oxygen (O_2_) concentration [9-11], which makes hypoxic tissue culture conditions beneficial. In particular, it has been shown that hypoxic MSC maintain significantly higher colony-forming unit capabilities and higher levels of stem cell-related genes [12]. They exhibit higher levels of osteoblastic and adipocytic differentiation markers (upon induction of the cells) as well as increased total protein levels compared to MSC cultured under 20% O_2 _(normoxic) conditions [13]. Several reports indicated that MSC cultured in hypoxia, demonstrate increased proliferation activity [13-16]. It has been speculated that oxygen concentration regulates the intricate balance between cellular proliferation and commitment towards differentiation, impacts 'stemness' of the MSCs [17]. Overall, it appears that the hypoxic environment is promoting a genetic program maintaining the undifferentiated and multipotent status of MSCs [12,18].

Migration to the sites of injury is an essential and characteristic feature of MSC [6-8] which is mediated by several regulators such as the Rho family of GTPases. In particular, the RhoA signaling cascade is believed to play an essential role in migration of MSC [19]. It is one of the best characterized members of the Rho family and has been shown to control cytoskeletal activation in many adherent cells, including MSC. RhoA regulates microtubule and actin assembly, the formation of stress fibers and cell adhesion, regulation of contraction and retraction. Therefore, the RhoA signaling cascade is believed to play an essential role in migration of MSC.

In this paper, we compared the *in vitro *directional migration of human MSC (hMSC) permanently cultured under normal (21%) and low O_2 _(5%) conditions (from now on called 'normoxic' and 'hypoxic', respectively). Recent reports demonstrated the increased motility of hMSC grown under hypoxic conditions compared to normoxic cells [20]. However, the directional migration of hMSC cultured in hypoxia has not been investigated. A series of soluble chemokines, growth factors and inflammatory cytokines were chosen as chemoattractants in the *in vitro *transmembrane migration assay. We found that hMSCs cultured in hypoxia have higher motility compared to the normoxic hMSCs towards most of the studied factors, particularly towards wound healing cytokines and cytokines found in ischemic brain and infarcted myocardium milieu. In an attempt to understand the underlying reasons for increased migration of hypoxic hMSC we measured RhoA activation in the cells. We discovered a markedly enhanced activation of RhoA in hypoxic MSC suggesting that high hMSC migration may occur via increased sensitivity to RhoA activation.

## Methods

### Cell culture

The mononuclear MSC fraction was isolated from a human bone marrow purchased from Lonza (the marrow was obtained from a healthy volunteer with appropriate informed consent and under ethical approval according to Lonza (Walkersville, MD, USA). The cells were cultured in tissue culture dishes (TCDs) in hMSC growth medium (hMSCGM): (D)MEM/F12+Glutamax™-I basal medium (LifeTechnologies, Grand Island, NY, USA) supplemented with 15% bovine growth serum (HyClone, Logan, UT, USA) and basic fibroblast growth factor (bFGF) (PeproTech, Rocky Hill, NJ, USA). For normoxic oxygen conditions (21% O2) cells were incubated in a standard humidified incubator at 37°C and 5% CO2. The hypoxia culturing was performed in a humidified 5% O2 and 5% CO2 incubator at 37°C.

### Cell characterization

#### Marker Expression

Cells were stained with appropriate antibodies conjugated to R-Phycoerythrin (PE) (BD Biosciences, San Jose, CA, USA) and expression scored by flow cytometry using standard protocols. The antibodies used were CD14, CD19, CD34, CD45, CD 73, CD90, CD105, CD166, HLA-DR and isotype control, diluted according to the manufacturers' recommendations.

#### Differentiation

For adipocyte and osteocyte differentiation, cells were seeded at high density and allowed to grow to confluence. The media was removed and replaced with appropriate differentiation media (Life Technologies). They were cultured further for two weeks with intermittent media changes. Adipocytes were visibly recognized as lipid droplets in cells. Osteocyte cultures were stained with Alizarin Red [21]. Chondrocyte differentiation was accomplished as a micromass, tissues were embedded and sectioned in a cryostat. The sections were fixed, washed and stained with Alcian Blue [22].

### Cytokine secretion

Passage 3 cells were seeded at 700 cells/cm^2 ^and cultured for seven to nine days in either normoxia or hypoxia, with intermittent media change. When the cultures reached approximately 70% confluence, media was replaced and cultures further incubated for 24 hours. Media supernatants were harvested and stored at -20°C. Cells were harvested and counted. Vascular endothelial growth factor (VEGF), stromal cell-derived factor-1α (SDF-1α) and angiopoietin-1 amounts in supernatants were determined by ELISA (R & D Systems, Minneapolis, MN, USA) and normalized to amounts secreted by one million cells.

### Colony Forming Unit - Fibroblasts (CFU-F)

Human bone marrow derived hMSCs were expanded under normal (normoxic) and 5% oxygen (hypoxic) conditions up to passage 2 and frozen in liquid nitrogen. A frozen stock of MSCs was thawed at 37°C, diluted with complete medium and recovered by centrifugation to remove dimethyl sulfoxide (DMSO). The cells were suspended in medium and plated at 100 cells per 9 cm TCD for CFU assay according to the following design: 1) normoxic hMSCs assessed under normoxic conditions, 2) normoxic cells assessed in the hypoxic environment, and 3) hypoxic cells assessed under hypoxic conditions (total of three experimental groups, five dishes per group). The cultures were stained 14 days later with Giemsa and the number of colonies (CFU-F) was manually counted. The experiment was repeated three times.

### Chemotaxis assay

A migration assay was performed in 48- or 96-well Corning Costar transwell chambers with porous polycarbonate membranes with a pore size of 5 um (Fisher Scientific, Rockville, MD, USA). hMSC at passage 4 were resuspended at 1 × 10^6^/mL in the migration medium supplemented with 1% BSA and insulin transferrin selenium (ITS) and seeded in the upper chamber. The following human recombinant proteins were used as chemoattractants in the lower compartment: hepatocyte growth factor (HGF), platelet-derived growth factor-AB (PDGF-AB), epidermal growth factor (EGF), VEGF-121, basic fibroblast growth factor (bFGF), insulin-like growth factor (IGF-1), macrophage inflammatory protein-3β (MIP-3β), macrophage inflammatory protein-1α (MIP-1α), B cell attracting chemokine-1 (BCA-1), regulation upon activation normal T cell express sequence (RANTES), growth regulated proteinα (GROα), fractalkine, SDF-1α, IL-1β, IL-6, IL-8, TNF-α (PeproTech). The concentration of each cytokine was optimized to ensure maximal cell migration through the porous membrane. The concentrations were: 40 ng/mL HGF, 10 ng/mL PDGF-AB, 10 ng/mL EGF, 10 ng/mL VEGF-121, 10 ng/mL bFGF, 30 ng/mL IGF-1, 10 ng/mL MIP-3β, 50 ng/mL MIP-1α, 5 ng/mL BCA-1, 150 ng/mL RANTES, 50 ng/mL GROα, 300 ng/mL Fractalkine, 150 ng/mL SDF-1α, 10 ng/mL IL-1β, 100 ng/mL IL-6, 50 ng/mL IL-8 and 50 ng/mL TNF-α. The chambers were incubated for four hours at 37°C in hypoxic or normoxic incubators. The cells from the top side of the membrane chamber were removed and the migrated cells on the underside of the membrane were completely dislodged by incubating the membrane inserts in 15 mM ethylenediaminetetraacetic acid (EDTA) for 20 minutes. All migratory cells were lysed with Triton X-100 and detected by CyQuant^® ^GR dye (Life Technologies) according to a standard protocol (Cell Biolabs, San Diego, CA, USA). Results were expressed as the mean number of net migrated cells over control cells (basal migration without chemotactic stimulus). Each condition was tested in four wells; each experiment was repeated four times.

### RhoA activation assay

Passage 2 cells were seeded at 2,000 cells/cm^2 ^and cultured for seven to nine days in either normoxic or hypoxic incubators, with intermittent media change. When the cultures reached approximately 70% confluence they were harvested and re-plated at the same concentration and cultured until reaching a confluence of 70% to 80%. For activation of RhoA, the growth media were changed to serum-free media containing either 100 ug/mL CN01 (calpeptin, Cytoskeleton, Denver, CO, USA) (activated MSC) or 1% DMSO (non-activated MSC), according to the manufacturer's instructions. After 10 minutes of incubation the cells were immediately put on ice and all consecutive manipulations were done on ice. To determine active RhoA (guanosine triphosphate (GTP)-bound) levels, a commercially available ELISA kit (G-LISA, Cytoskeleton) was utilized on the whole cell extracts according to the manufacturer's instructions. The results were normalized to the total protein level. Each experiment was repeated four times in 15 cm TCDs, two dishes per experimental group.

### Hypoxia inducible factor -1 activity assay

Passage 2 cells were seeded at 2,000 cells/cm^2 ^and cultured for seven to nine days in either normoxic or hypoxic incubators with intermittent media change. When the cultures reached approximately 70% confluence they were harvested and re-plated at the same concentration and cultured until reaching a confluence of 70% to 80%. After that time, cells were immediately put on ice and the nuclear extracts were collected according to the manufacturer's protocol (Nuclear Extract Kit, Active Motif, Carlsbad, CA, USA). To determine activated hypoxia inducible factor-1 (HIF-1) levels, a commercially available ELISA kit (Active Motif) was utilized on the nuclear extracts according to the manufacturer's instructions. The results were normalized to the number of cells. Each experiment was performed four times in 15 cm TCDs, three dishes per experimental group. The specificity of the assay was confirmed by using the wild-type consensus oligonucleotide which competes for HIF-1 binding and the mutated consensus oligonucleotide, which does not bind HIF-1.

### Statistics

Data are representative of three or more independent experiments and presented as mean ± SEM. Statistical significance was assessed using Student's unpaired t-Tests (Microsoft Excel). Probability values of less than 0.05 were considered significant.

## Results

### Cell characterization and differentiation, VEGF production

Both normoxia and hypoxia cultured hMSC stained positively for CD73, CD90 and CD105, a set of markers required to be expressed according to minimal criteria for defining multipotent MSC adopted by the International Society for Cell Therapy [23]. Also, both cells types did not express hematopoietic markers CD14 (monocytes/macrophages), CD19 (B cells), CD34 (progenitors/endothelial cells) or CD45 (leukocytes), or HLA-DR. Flow cytometry analysis revealed no difference in the expression of surface markers between hypoxic and normoxic hMSC. The fraction of HLA-DR-positive cells never exceeded 2%. Cell cultures grown in hypoxia and/or normoxia differentiated into adipocytes, osteocytes and chondrocytes and secreted a number of cytokines. hMSCs grown in hypoxia secreted greater amounts of some cytokines such as VEGF, SDF-1, and angiopoietin-1 (data not shown).

### Colony Forming Unit - Fibroblasts

**Figure 1:**
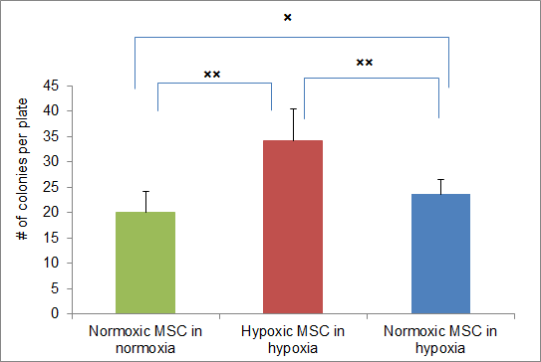
**Clonogenicity of bone marrow derived hMSC determined in CFU-F assay**. hMSCs grown and assayed under normal O_2 _(21%) - Normoxic MSC in normoxia, grown and assayed in low O_2 _(5%) - Hypoxic MSC in hypoxia, grown in normoxia and assayed in hypoxia - Normoxic MSC in hypoxia. The assay was performed in 9 cm tissue culture dishes, five dishes per experimental group. The cultures were terminated 14 days after initiation. The dishes were stained with Giemsa and the colonies (CFU-F) were manually counted. Each experiment was repeated three times. Columns represent the average number of colonies per plate from all three experiments combined (*n *= 15). The bars represent 1 SEM. × - *P *< 0.05, ×× - *P *< 0.0001. CFU-F, colony forming unit fibroblasts; hMSC, human mesenchymal stem cells; SEM, standard error of the mean.

The effect of hypoxia on clonogenicity of hMSC was evaluated by the CFU-F assay. Hypoxic and normoxic hMSC were seeded in TCDs at a density of 1.66 cells/cm^2 ^and incubated for 14 days. Initially, the assay was performed in the same oxygen environment as the original cell culture. The results showed dramatically higher CFU-F numbers (1.7 fold difference) produced by hypoxic hMSC (Figure 1).

It has been shown that MSC demonstrate higher CFU-F when placed in hypoxic conditions [13,24,25]. Therefore, to rule out the effect of the hypoxic assay environment, the normoxic cells were assessed for colony formation under hypoxic conditions. We observed a 20% increase in the number of colonies produced by normoxic hMSC in hypoxia compared to a normoxic environment. However, the number remained 50% (*P *= 4 × 10^-6^) lower than long term cultured hypoxic hMSC.

These data confirmed the notion that hMSC grown permanently in hypoxia have higher clonogenic potential compared to normoxic cells. It is unlikely that the observed phenomenon can be explained by hypoxic conditions during the assay. It may rather reflect the increased number of immature stem cells in the population of hypoxic hMSC.

### Migration

Next, we studied the migration potential of hypoxic and normoxic hMSCs *in vitro*, using a transmembrane assay. Three groups of molecules were used as chemoattractants: growth factors (HGF, PDGF-AB, EGF, VEGF-121, bFGF, IGF-1), chemokines (MIP-3β, MIP-1α, BCA-1, RANTES, GROα, Fractalkine, SDF-1α), and inflammatory cytokines (IL-1β, IL-6, IL-8, TNF-α). The assay was performed in the same oxygen environment as the original cell culture.

**Figure 2:**
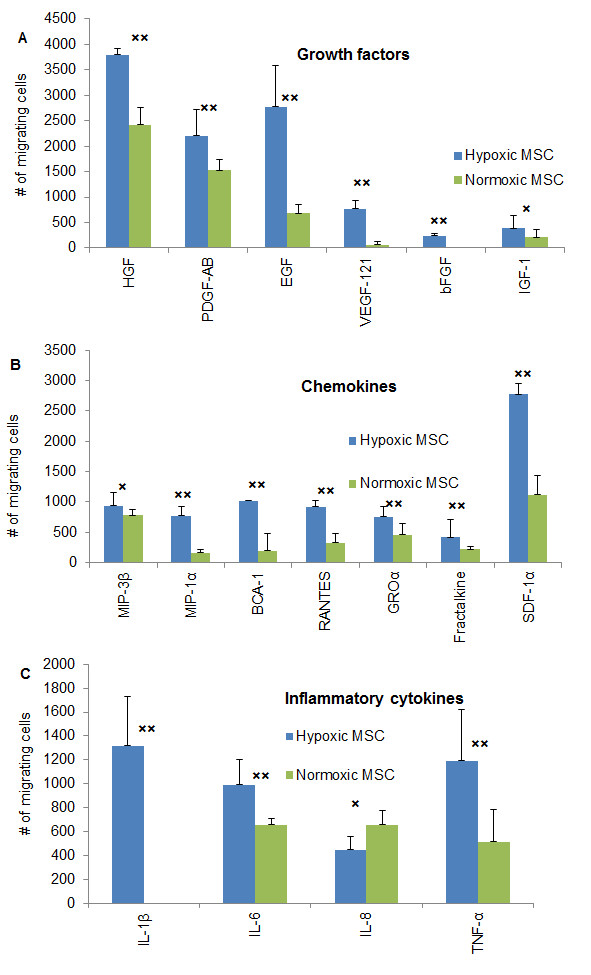
**Migration capacity of hypoxic and normoxic hMSCs towards growth factors, chemokines and inflammatory cytokines**. *In vitro *migration was assayed in transwell plates with porous polycarbonate membranes (pore size: 5 um). Columns represent the difference between the mean numbers of induced and spontaneously migrating cells (no chemoattractant added). Each condition was tested in four wells; each experiment was repeated four times. The bars represent 1 SEM from four independent experiments combined (*n *= 16). × - *P *> 0.05, ×× - *P *< 0.05. (**A**): Migration of (BM) hMSC towards growth factors, (**B**): Migration of (BM) hMSC towards chemokines, (**C**): Migration of (BM) hMSC towards inflammatory cytokines. hMSC, human mesenchymal stem cells; SEM, standard error of the mean.

Figure 2 (A, B, C) demonstrates that for the most part the number of migrating hypoxic hMSC was significantly higher compared to normoxic cells (HGF, EGF, VEGF-121, bFGF, BCA-1, RANTES, SDF-1α, IL-1β, IL-6, TNF-α, *P *< < 0.05), It should be noted that IL-8 was the only cytokine that showed some inhibitory effect on migration of hypoxic hMSC, although the difference did not reach significance between the oxygen groups (*P *= 0.87). Also, significance was not reached in the case of IGF-1 and MIP-3β (*P *= 0.21 and 0.25, respectively). However, all three cytokines (IL-8. IGF-1 and MIP-3β) stimulated a statistically significant increase in migration over the base line in both hypoxic and normoxic hMSC.

It is interesting to observe that the migration towards the growth factors was more prominent compared to inflammatory cytokines and chemokines. On average, 280% more cells migrated towards HGF, PDGF-AB and EGF than towards inflammatory cytokines and chemokines. SDF-1α demonstrated the highest chemotactic activity among them, being as stimulating for cell migration as the most active growth factors.

To rule out the effect of the assay environment on migration of hMSC, the normoxic cells were transferred for the assay into hypoxia. Although we observed more migrating cells in normoxic hMSC under hypoxic conditions compared to normoxia, the numbers remained significantly lower (*P *< 0.05) than in long term cultured hypoxic hMSC for most of the cytokines tested except for PDGF-AB, MIP-1α, BCA-1 and GROα (*P *> 0.05, data not shown).

In addition, we found that the increased migration of hypoxic cells was particularly noticeable toward cytokines involved in the wound healing process such as EGF, bFGF, VEGF-121, IL-1β, IL-6 and TNF-α (Figure 2). All of them have been shown to participate in a complex signaling network leading to wound repair and regeneration [26].

Taken together, our data suggest that the *in vitro *migration of hMSCs permanently grown in hypoxia is significantly higher compared to hMSC grown under normoxic conditions.

### Evaluation of mechanisms involved in the enhanced hypoxic hMSC migration

#### Role of RhoA in hMSC migration

First, we explored the involvement of RhoA in the observed phenomena. As has been already mentioned in the Introduction, the RhoA signaling cascade is believed to play an essential role in the migration of MSC [19]. It controls cytoskeletal activation in many cell types including MSC [27,28]. To become functional RhoA needs to be activated by a variety of growth factors, cytokines, adhesion molecules, hormones, integrins, G-proteins and other biologically active substances [29]. *In vitro *it can be activated by synthetic molecules, such as CN01 (calpeptin), which was used in this study. We measured the concentration of GTP-bound (active) RhoA in the whole cell extracts of activated hypoxic and normoxic hMSC. The activation of the hMSC for the assay was performed in the same oxygen environment as the original cell culture.

**Figure 3:**
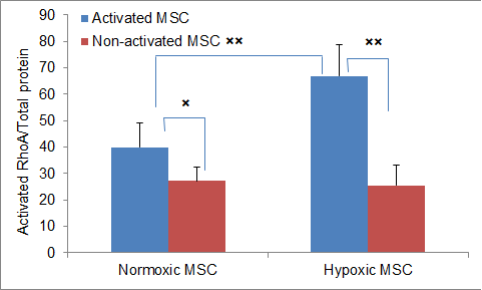
**RhoA activation by calpeptin measured by G-LISA in hypoxic and normoxic hMSCs**. The activation was performed in the same oxygen environment as the original cell culture. Cells were 80% to 90% confluent when treated with 0.1 mg/mL of calpeptin for 10 minutes, then lysed and the lysates were subjected to the G-LISA assay (activated MSC). The control cells were treated with the media, which did not contain calpeptin (non-activated MSC). The assay was performed in two dishes per experimental group; each experiment was repeated four times. Data are presented with the subtracted background (medium alone). Columns represent the average of four experiments combined (*n *= 8). The bars represent 1 SEM. × - *P *< 0.05, ×× - *P *< 0.01. MSC, mesenchymal stem cells; SEM, standard error of the mean.

The results showed that upon activation of hMSC with CN01 the level of GTP-bound RhoA in hypoxic cells is significantly higher (1.7-fold increase) than in normoxic cells (Figure 3). It is noteworthy that the level of GTP-bound RhoA in non-activated hMSC was not different between the groups (*P *= 0.66), suggesting that long term cultivation in hypoxia enhances activation of RhoA in hMSC.

Overall, these data, together with the observed increased migration of hypoxic hMSC, support the involvement of RhoA in hMSC migration. Moreover, we speculate that hypoxia may lead to greater hMSCs migration via increased sensitivity to RhoA activation.

#### Active HIF-1 and RhoA activation in hypoxic hMSC

To study how hypoxia may lead to enhanced RhoA activation we assayed the activity of HIF-1 in hypoxic and normoxic hMSCs. It has been established that hypoxia-activated HIF-1 is a major contributor to cell transcription alteration [30]. Activated HIF-1 was shown to be a potential upstream regulator of RhoA activation in MSC [19,31].

In this study, we measured HIF-1 activity in hypoxic and normoxic cells using the TransAM method (Activ Motif). This approach allows detecting only activated HIF-1 in cell nuclear extracts. The specificity of the assay was confirmed by adding either the wild-type or mutated consensus oligonucleotides to the tested samples. The addition of the wild-type nucleotide to the tested samples completely suppressed HIF-1 binding to the probe immobilized on the assay plate. Conversely, the addition of the mutated oligonucleotide did not affect the assay results, thus confirming the specificity of the testing method.

**Figure 4:**
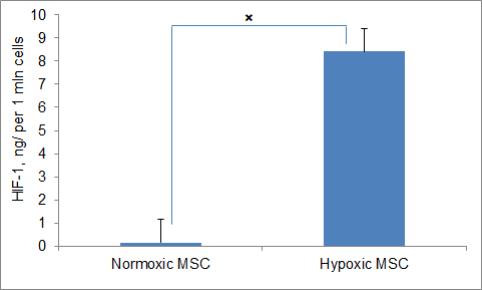
**Activated HIF-1 measured by TransAM HIF-1 ELISA in hypoxic and normoxic hMSCs**. Columns represent mean amounts of activated HIF-1 normalized to amounts per million harvested cells. The assay was performed in three dishes per experimental group; each experiment was repeated four times. Data are presented with the subtracted background (medium alone). Columns represent the average of four experiments combined (*n *= 12). The bars represent 1 SEM. ×- *P *< < 0.01. ELISA, enzyme-linked immunosorbent assay; HIF-1, hypoxia inducible factor-1; hMSCs, human mesenchymal stem cells; SEM, standard error of the mean.

We found that normoxic hMSCs demonstrate very low (undetectable) levels of active HIF-1, unlike hypoxic cells which have accumulated large amounts of this factor (8.4 ng per 1 million cells) (Figure 4). The data suggest that hypoxia-activated HIF-1 may be involved in enhanced activation of RhoA in hypoxic hMSC. Thus, it can be speculated that high activity of HIF-1 in hypoxic hMSC leads to increased cell motility via the stimulated activation of RhoA.

## Discussion

In this paper, we provide the evidence for higher *in vitro *targeted migration of hMSC cultured under hypoxic conditions compared to the cells grown in normal oxygen toward most of the tested cytokines. Previous studies showed the enhancing effect of short term exposure to hypoxia on migration of MSC [15,19,28,32-34]; an increase in cell velocity and Euclidian distance on a series of matrices for the hMSC cultured in 2% hypoxia has also been shown [20]. To our knowledge we are the first to report higher targeted migration of hMSC grown in hypoxia for the entire culturing period.

We tested migration toward the factors from three major groups - growth factors, chemokines and inflammatory cytokines - since hMSCs have been shown to express an array of functional receptors for those molecules [35-38]. We found that all 17 cytokines tested in this study mediated *in vitro *cell migration of hMSC, with the least activity among chemokines and the most among growth factors, which is in agreement with other studies [35-37].

In addition, we noticed that hypoxic cells are more responsive to cytokines often present in a wound milieu (Figure 2). The most important among them are the EGF family, the fibroblast growth factor family, VEGF-121, PDGF-AB, the IL family and TNF-α [26]. The enhanced migration of hMSCs toward the wound healing cytokines can potentially accelerate the formation of new tissue and ultimately wound closure.

In addition, we found a significantly better migration of hypoxic cells toward such factors as HGF, SDF-1α, the IL family cytokines and TNF-α. It is tempting to speculate that this feature may lead to better *in vivo *homing of long term hypoxia-cultured hMSCs to the sites of infarct and stroke since those factors are abundant at the sites of ischemic injury [39-43].

It is noteworthy that migration of normoxic hMSC transferred for the assay into a hypoxic environment was still lower than in hypoxic cells, but higher than in normoxic cells assessed in normoxia (data not shown) confirming the known stimulating effect of acute hypoxia on the migration of MSC. It has been shown that hypoxic pre-conditioning increases MSC migration [32] due, at least in part, to up-regulated expression of the chemokine receptors CX3CR1 and CXCR4 [15], as well as the c-Met receptor for HGF [33]. It is possible that the increased migration of cells permanently cultured in hypoxia can be explained by up-regulation of a series of receptors. However, we believe that the major reason for the mentioned phenomenon is the undifferentiated status of hypoxic MSC.

The latter notion is supported by the analysis of CFU-F efficiency, which demonstrated that hypoxic hMSC have increased CFU-F, compared to similar cells grown under normoxic conditions (Figure 1). Normoxic cells assayed for CFU-F in hypoxia still produced a significantly lower number of colonies compared to hypoxic cells ruling out the effect of assay environment. Rather, the higher number of CFU-F in hypoxic cells may be due to the accumulation of immature stem cell progenitors as previously proposed [12].

Perhaps the most significant finding of this study is the enhanced activation of RhoA in hypoxic hMSC. The RhoA signaling cascade is believed to play an essential role in the migration of MSC [19]. The family of RhoGTPases directs a variety of cell responses including cell migration, adhesion, transcription, growth and so on [44]. It controls cytoskeletal activation in many cell types including MSC [27,28]. We demonstrated that upon activation with CN01 the increase of GTP-bound RhoA over non-activated cells is considerably higher in hypoxic hMSC compared to normoxic cells (3-fold and 1.5-fold, respectively). It remains to be investigated whether this phenomenon is true for other types of stem cells reflecting their undifferentiated status. The precise mechanism of the observed difference in sensitivity is unclear. Recently, it has been shown that HIF-1 and RhoA cross-talk suggesting the role of HIF-1 in hypoxic regulation of MSC migration through the RhoA signaling cascade [19,31].

HIF-1 is one of the key regulators of oxygen homeostasis [45]. HIF-1 is a heterodimeric protein that consists of two subunits, HIF-1α and HIF-1β. While HIF-1β is constitutively expressed, the expression of HIF-1α is lower in normoxic conditions (O_2 _> 6%) due to its hydroxylation and degradation. In hypoxia (O_2 _< 6%) both subunits, HIF-1α and β, survive degradation and form an active HIF-1 heterodimer, which binds to the HRE in the cell nucleus [46]. It has been shown that artificial stabilization of HIF-1 in normoxia by desferrioxamine (DFO), as well as pre-conditioning in 1% hypoxia result in attenuation of RhoA activation [19,31] suggesting that activated HIF-1 is a potential upstream regulator of RhoA. We found that both activated HIF-1 and GTP-bound RhoA were elevated in long term hypoxic cells, reflecting a strong link between HIF-1 and activation of RhoA in hypoxic hMSC.

It should be noted that the RhoA activation pattern observed by Raheja *et al. *[19] for hMSC pre-conditioned in 1% hypoxia or treated with DFO (state close to anoxia) is different compared to the data presented in this paper. It can be due to the fact that hypoxia pre-conditioning, which reflects the acute cellular response to hypoxia [33] rather than cell differentiation status, is substantively different from permanent cell cultivation in the low O_2 _environment. Also, 0%/1% and 5% oxygen concentrations may potentially have different effects on MSCs physiology [18]. Therefore, we believe it is difficult to directly compare our data to the results reported by Raheja *et al. *[19].

## Conclusions

In conclusion, in this paper we confirmed the notion that hMSC cultured under hypoxic conditions preserve their undifferentiated status as judged by higher colony-forming potential and increased *in vitro *targeted migration. In addition, for the first time, we demonstrated that hypoxic MSC compared to normoxic are dramatically more sensitive to activation of RhoA which may contribute to the observed high migration. Finally, we found that hypoxic hMSCs migrate better towards wound healing cytokines and cytokines found in an ischemic tissue milieu suggesting their potential therapeutic advantages over normoxic cells.

## Abbreviations

BCA-1: B cell attracting chemokine-1; bFGF: basic fibroblast growth factor; BSA: bovine serum albumin; CFU-F: colony forming unit fibroblasts; DFO: desferrioxamine; (D)MEM: (Dulbecco's) modified Eagle's medium; DMSO: dimethyl sulfoxide; EGF: epidermal growth factor; ELISA: enzyme-linked immunosorbent assay; GROα: growth regulated protein α; GTP: guanosine triphosphate; HGF: hepatocyte growth factor; HIF-1: hypoxia inducible factor-1; hMSC: human bone marrow derived mesenchymal stem cells; hMSCGM: hMSC growth medium; HRE: hypoxia response element; IGF-1: insulin-like growth factor; IL-1β: interleukin-1β; IL-6: interleukin-6; IL-8: interleukin-8; ITS: insulin transferrin selenium; MIP-1α: macrophage inflammatory protein-1α; MIP-3β: macrophage inflammatory protein-3β; PDGF-AB: platelet-derived growth factor-AB; RANTES: regulation upon activation normal T cell express sequence; SDF-1α: stromal cell-derived factor-1α; TCD: tissue culture dishes; TNF-α: tumor necrosis factor- α; VEGF-121: vascular endothelial growth factor-121.

## Competing interests

GV, LK, MGM and AK are employees of 'Stemedica' Cell Technologies. GS and TT are uncompensated interns.

## Authors' contributions

GV, LK, MGK, GS and TT performed experiments, assembled and collected the data. MGM and LK cultured the cells and performed the CFU-F assay. GV, GS and TT carried out the transmembrane migration assays. GV and GS performed the ELISA analyses. GV and AK contributed to the conception and design of the study, interpretation of the results and manuscript writing. All authors read and approved the final manuscript.
